# Differential Characteristics and Prognosis of PD-L1–Positive Endometrial Carcinomas: A Retrospective Chart Review

**DOI:** 10.3390/life11101047

**Published:** 2021-10-06

**Authors:** Justin Z. Amarin, Razan Mansour, Sura Al-Ghnimat, Maysa Al-Hussaini

**Affiliations:** 1 Office of Scientific Affairs and Research, King Hussein Cancer Center, Amman 11941, Jordan; justinzamarin@gmail.com (J.Z.A.); razanamansour@gmail.com (R.M.); 2 Department of Pathology and Laboratory Medicine, King Hussein Cancer Center, Amman 11941, Jordan; SA.12608@khcc.jo

**Keywords:** DNA mismatch repair, endometrial carcinoma, immunohistochemistry, programmed cell death 1 ligand 1 (PD-L1)

## Abstract

Women with endometrial carcinomas that express PD-L1 may respond better to immunotherapy. Our aim was to investigate the differential characteristics of PD-L1–positive endometrial carcinomas and the prognostic significance of PD-L1. We performed a retrospective chart review of 231 women with endometrial carcinomas who were managed at King Hussein Cancer Center (2007–2016) and performed immunohistochemistry for MLH1, PMS2, MSH2, MSH6, p53, and PD-L1. Overall, 89 cases (38.5%) were MMR-deficient. PD-L1 was expressed in 49 cases (21.2%) and its expression was significantly associated with MLH1/PMS2 deficiency (*p* = 0.044) but not MSH2/MSH6 deficiency (*p* = 0.59). p53 was mutant in 106 cases (46.5%), and its mutation was significantly associated with MMR proficiency (*p* < 0.001) but not PD-L1 expression (*p* = 0.78). In women with endometrioid adenocarcinomas, PD-L1 expression was significantly associated with the Fédération Internationale de Gynécologie et d′Obstétrique (FIGO) grade (*p* = 0.008). Overall, PD-L1 expression did not significantly predict overall survival in unadjusted or adjusted analyses (*p* = 0.92 and 0.54, respectively). In conclusion, tumors with MLH1/PMS2 loss and high-grade endometrioid adenocarcinomas were more likely to express PD-L1 in tumor cells. Further research is required to investigate whether the presence of either characteristic signals a higher likelihood of a favorable response if immunotherapy is administered.

## 1. Introduction

Interactions between T cells and antigens are regulated by co-stimulatory and co-inhibitory signals—the immune checkpoints. One of the key immune checkpoints is the dyad of programmed cell death protein 1 (PD-1) and its ligand, PD-1 ligand 1 (PD-L1). PD-1 is a co-inhibitory transmembrane receptor expressed by T cells. By engaging PD-1, PD-L1 can inhibit the proliferation, survival, and cytokine production of T cells [1,2]. Tumor cells that express PD-L1 can use the PD-1/PD-L1 immune checkpoint to evade the immune system [3].

The PD-1/PD-L1 immune checkpoint is of major clinical significance because antibody-based inhibitors can improve the outcomes of patients with diverse advanced cancers—especially patients with PD-L1–positive tumors [2]. Albeit with limitations, PD-L1 expression in tumor and/or immune cells is used to predict response to immunotherapy, and it is the most common immune-based biomarker in current clinical practice [4]. Many tissue-based biomarker assays are currently in use to estimate the response to immunotherapy, including PD-L1 immunohistochemistry (IHC), tumor mutational burden, gene expression profiling, and multiplex IHC/immunofluorescence. Though other biomarker assays perform better, PD-L1 IHC is the most well-established biomarker for response to immunotherapy [5].

Endometrial cancer is the most common gynecologic cancer in the United States. The mainstay of treatment for endometrial cancer is surgery, with adjuvant radiotherapy and/or chemotherapy as indicated. Recently, immunotherapy has been the focus of several investigations [6]. Indeed, many clinical trials of immunotherapy for endometrial cancer are ongoing, and preliminary results are promising [7]. For example, pembrolizumab, an anti–PD-1 monoclonal antibody, demonstrated durable antitumor activity in a cohort of women with PD-L1–positive, advanced endometrial cancer [8]. In addition, women with mismatch repair (MMR)-deficient, advanced or recurrent endometrial cancer responded to avelumab, an anti-PD-L1 monoclonal antibody, regardless of PD-L1 status [9]. Interestingly, atezolizumab, another anti–PD-L1 monoclonal antibody, showed durable clinical benefit in some women with advanced or recurrent endometrial cancer, and the clinical benefit appeared to increase with higher PD-L1 expression [10].

Given that PD-L1 expression may predict the response to immunotherapy in endometrial cancer, our primary objective was to compare the clinicopathologic characteristics of PD-L1–positive tumors with those of PD-L1–negative tumors. We hypothesize that any differential clinicopathologic characteristics may be candidate predictors of the response to immunotherapy, to be investigated in follow-up studies. Our secondary objective was to investigate the prognostic significance of clinicopathologic characteristics in endometrial cancer, with an emphasis on the independent prognostic significance of PD-L1 expression.

## 2. Materials and Methods

### 2.1. Design, Setting, and Participants

We performed a retrospective chart review of women with endometrial carcinomas who were managed at King Hussein Cancer Center between 2007 and 2016. King Hussein Cancer Center is the only comprehensive cancer center in Jordan and serves roughly 60% of all patients with cancer in Jordan [11]. We included all women with a histologically confirmed endometrial carcinoma who were registered in the institutional Cancer Registry (established July 2006) in the mentioned years. The number of cases in the Cancer Registry with sufficient tissue material for further immunohistochemistry determined the sample size. We retrieved the following data from the registry: date of birth, date of definitive surgery, nationality, family history of cancer, histopathology, Fédération Internationale de Gynécologie et d′Obstétrique (FIGO) grade (for endometrioid adenocarcinomas), chemotherapy, radiotherapy, FIGO stage, distant metastasis, relapse, overall survival (OS) status, date of last follow-up, and date of death. We defined OS as the length of time from the date of definitive surgery to the date of last follow-up or the date of death from any cause. We accessed electronic medical records to supplement missing data. We also supplemented survival data with data from the national Civil Status and Passports Department, which is a branch of government whose duties include curating current survival data. The study was reviewed and approved by the Institutional Review Board (IRB) of King Hussein Cancer Center (Amman, Jordan; 17KHCC107; approved 20 November 2017). The IRB complies with the Declaration of Helsinki and the Good Clinical Practice (GCP) guidelines. The requirement for informed consent was waived by the IRB because the work involves existing data and specimens.

### 2.2. Immunohistochemistry

We used the BenchMark ULTRA IHC/ISH System (Ventana Medical Systems, Tucson, AZ, USA) to perform IHC for four MMR proteins (MLH1, PMS2, MSH2, and MSH6), p53, and PD-L1 (Table 1). We performed IHC for MLH1 and MSH2 only if the nuclear expressions of PMS2 and MSH6, respectively, were lost. We ran the appropriate external positive and negative controls with each antibody. For the MMR proteins, we defined loss-of-expression as loss of nuclear staining in the presence of the appropriate positive internal control. Further, we defined MMR deficiency as loss-of-expression of any one or combination of the four MMR proteins. We classified tumors as p53-mutant if tumor cells showed no staining (null staining) or ≥80% showed diffuse strong nuclear staining. For PD-L1, we measured the percentage of tumor cells (tumor proportion score, TPS) with any membranous staining, and we defined positivity on the basis of a cutoff value ≥1%.

### 2.3. Statistical Methods

We used R (version 4.0.2, R Core Team, Vienna, Austria) to perform data analyses. First, we computed summary statistics to describe the clinical and tumoral characteristics of the full sample (absolute frequencies and percentages for categorical data and means and standard deviations for continuous data). We then stratified the women according to tumoral PD-L1 TPS (<1% or ≥1%), described the clinical and tumoral characteristics of the strata, and tested associations using Pearson’s *χ*^2^ test. We followed up the main analyses with ancillary analyses to characterize the relationship between variables that were associated with PD-L1 expression. We also used the Kaplan–Meier method to plot OS curves stratified by the clinicopathologic characteristics of the women. We then estimated the 1-and 5-year OS rates and compared the curves using the log-rank test. Finally, we fit a Cox regression model and included as predictors nine clinicopathologic variables, which we had selected on the basis of a literature search and expert knowledge. For all hypothesis tests, we interpreted values of *p* ≤ 0.05 to indicate statistical significance.

## 3. Results

**Figure 1:**
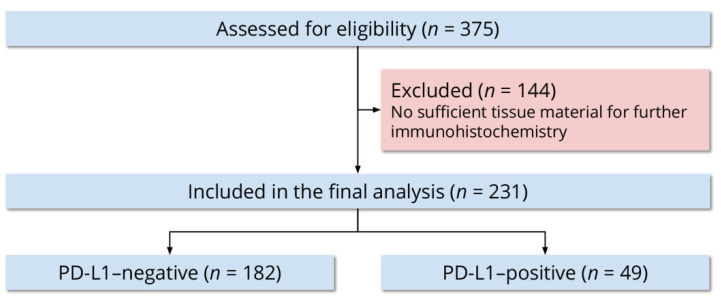
Flow diagram of the study participants.

Out of 375 women, 231 (61.6%) had sufficient tissue material for further immunohistochemistry. We included all 231 women in the final analysis (Figure 1). The mean age at definitive surgery was 61 ± 11 years. Of all women, 139 (60.2%) were 65 years or younger and 92 (39.8%) were older than 65 years. Two hundred women (86.6%) were nationals of Jordan and 31 (13.4%) were nationals of other countries in the Middle East and North Africa. Forty-five women (19.5%) reported a family history of cancer in a first-degree relative, 10 (4.3%) reported a family history of cancer in a second-degree relative, 133 (57.6%) reported no family history of cancer, and 43 (18.6%) were unable to provide a family history.

We studied the clinical and tumoral characteristics of the women. All 231 women underwent definitive surgery, 24 (10.4%) underwent a follow-up surgery, and one (0.4%) underwent a third surgery. Overall, 65 women (28.1%) received chemotherapy and 170 (73.6%) received radiotherapy. The FIGO stage was I in 121 women (52.4%), II in 46 (19.9%), III in 42 (18.2%), IV in 21 (9.1%), and undocumented in one (0.4%). Distant metastases were documented in 32 women (13.9%). By histopathology, 156 tumors (67.5%) were endometrioid adenocarcinomas, 33 (14.3%) were serous carcinomas, 17 (7.4%) were carcinosarcomas, 11 (4.8%) were undifferentiated/dedifferentiated carcinomas, 10 (4.3%) were mixed cell adenocarcinomas, and four (1.7%) were clear cell carcinomas. Of the mixed cell adenocarcinomas, six (60%) were serous and endometrioid, two (20%) were serous and clear, and two (20%) were endometrioid and clear. According to the Bokhman classification, 156 tumors (67.5%) were type I and 75 (32.5%) were type II. Of 156 endometrioid adenocarcinomas, 52 (33.3%) were FIGO grade I, 83 (53.2%) were FIGO grade II, and 21 (13.5%) were FIGO grade III.

**Figure 2:**
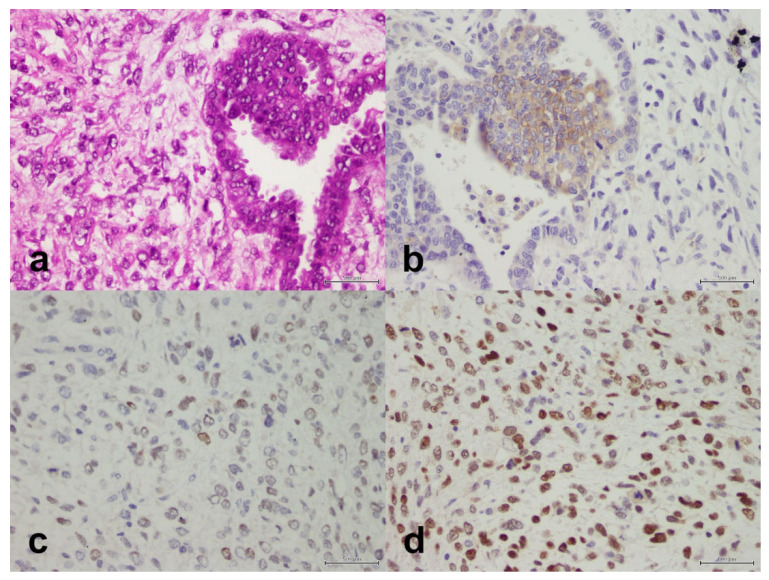
Carcinosarcoma composed of a component of high-grade serous carcinoma and a component of malignant mesenchymal stroma. (**a**) H&E, X40. (**b**) PD-L1 is positive in the epithelial component only, X40. (**c**) PMS2 is retained, X40. (**d**) MSH6 is retained, X40. Scale bar: 500 μm.

**Figure 3:**
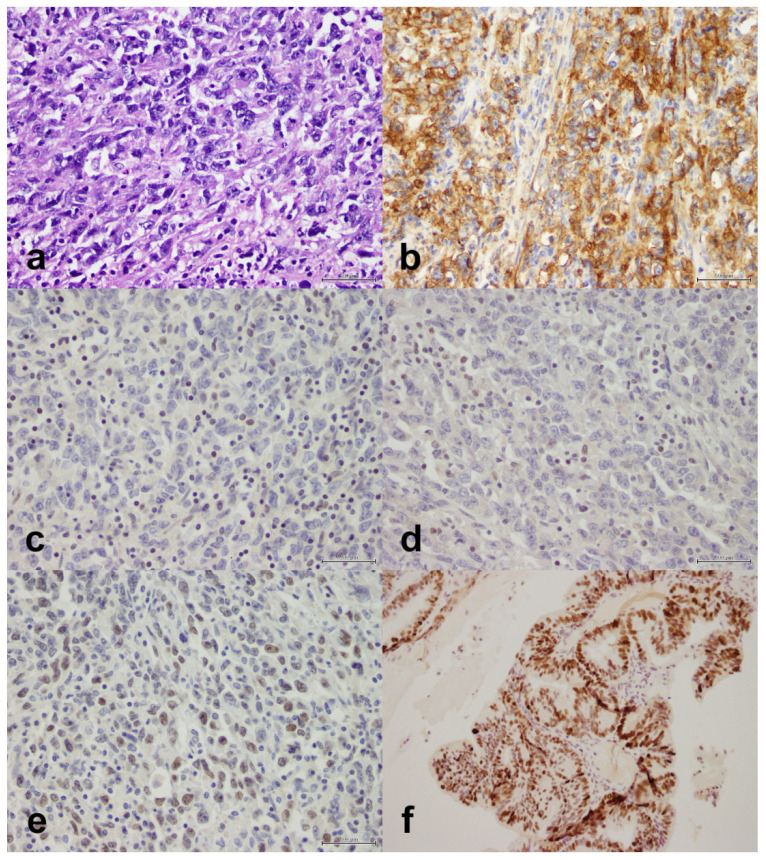
Undifferentiated endometrial carcinoma with sheets of cells, some of which show rhabdoid morphology. (**a**) H&E, X40. (**b**) PD-L1 is positive in 70% of tumor cells, X40. (**c**) MLH1 is lost (lymphocytes are an internal positive control), X40. (**d**) PMS2 is lost (lymphocytes are an internal positive control), X40. (**e**) MSH6 is retained, X40. (**f**) Mutant p53, X40. Scale bar: 500 μm.

**Figure 4:**
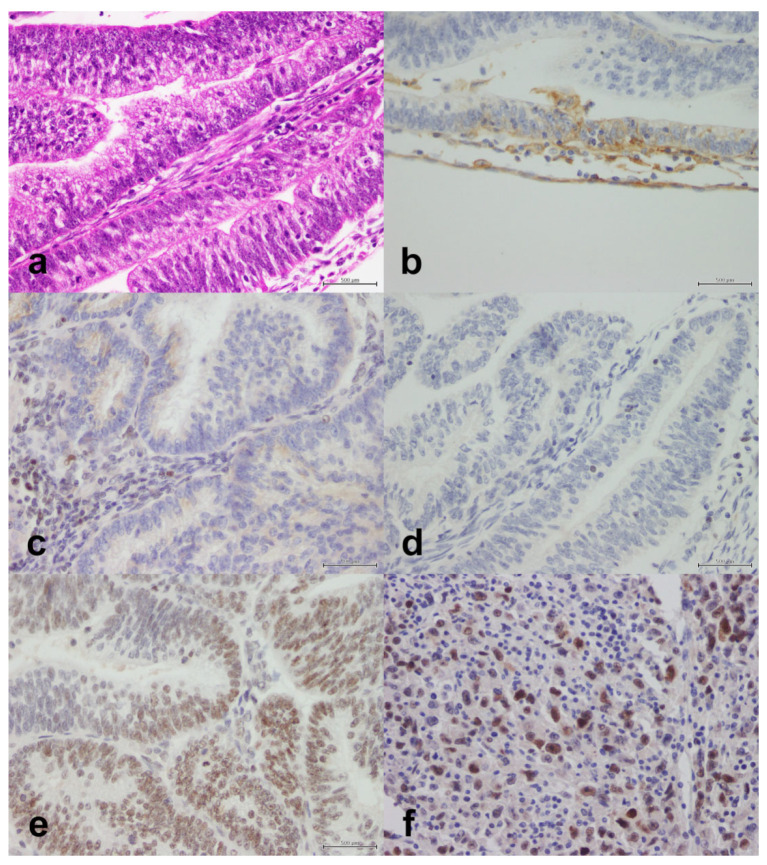
Endometrioid adenocarcinoma with moderate-to-poor differentiation. (**a**) H&E, X40. (**b**) PD-L1 is positive in 1% of tumor cells, X40. (**c**) MSH2 is lost (lymphocytes are an internal positive control), X40. (**d**) MSH6 is lost (lymphocytes are an internal positive control), X40. (**e**) PMS2 is retained, X40. (**f**) Mutant p53, X40. Scale bar: 500 μm.

We studied the expression of MLH1/PMS2, MSH2/MSH6, and p53 (Figure 2, Figure 3 and Figure 4). Overall, 89 cases (38.5%) were MMR-deficient and 142 (61.5%) were MMR-proficient. MLH1 and PMS2 were concomitantly lost in 55 cases (23.8%) and PMS2 alone was lost in nine cases (3.9%). MSH2 and MSH6 were concomitantly lost in 15 cases (6.5%) and MSH6 alone was lost in 19 cases (8.2%). PMS2 and MSH6 were concomitantly lost in nine cases (3.9%). Overall, p53 was mutant in 106 cases (46.5%) and wild-type in 122 (53.5%). p53 mutation occurred more commonly in MMR-proficient cases (*n* = 86, 61.4%) than in MMR-deficient cases (*n* = 20, 22.7%), and the association was significant (*p* < 0.001).

We studied the expression of PD-L1 (Figure 2, Figure 3 and Figure 4). The protein was expressed in 49 cases (21.2%). The clinical and tumoral characteristics of the study population stratified by PD-L1 expression are outlined in Table 2. PD-L1 expression was not significantly associated with the age group at definitive surgery (*p* = 0.87), nationality (*p* = 0.84), family history (*p* = 0.98), histopathology (*p* = 0.32), Bokhman type (*p* = 0.29), chemotherapy (*p* = 0.062), radiotherapy (*p* = 0.28), FIGO stage (*p* = 0.78), distant metastasis (*p* = 0.57), relapse (*p* = 0.96), isolated PMS2 expression (*p* = 0.94), MSH2/MSH6 expression (*p* = 0.59), isolated MSH6 expression (*p* = 0.55), MMR status (*p* = 0.090), or p53 status (*p* = 0.78). However, PD-L1 expression was significantly associated with MLH1/PMS2 expression (*p* = 0.044). In women with endometrioid adenocarcinomas, PD-L1 expression was significantly associated with FIGO grade in endometrioid adenocarcinoma (*p* = 0.008); PD-L1 was expressed in six of 52 grade I tumors (11.5%), 15 of 83 grade II tumors (18.1%), and nine of 21 grade III tumors (42.9%). In the same subset of women, MLH1/PMS2 expression was not significantly associated with FIGO grade (*p* = 0.42). Of six MLH1/PMS2–deficient grade III endometrioid adenocarcinomas, PD-L1 was expressed in four (66.7%).

Finally, we performed a survival analysis using the Kaplan–Meier method and Cox regression (Table 3). The total follow-up time for the full cohort was 1350.8 years (median, 5.6 years; mean, 5.8 years). During the follow-up period, 92 women (39.8%) died. The 1- and 5-year OS rates for the full cohort were 88.3% (95% CI, 84.3%–92.6%) and 64.3% (95% CI, 58.4%–70.8%), respectively. The prognostic significance of all nine candidate predictors is summarized in Table 3. Notably, PD-L1 expression did not significantly predict OS in unadjusted or adjusted analyses (*p* = 0.92 and 0.54, respectively).

## 4. Discussion

We compared the clinical and tumoral characteristics of 49 women with PD-L1–positive endometrial carcinomas and 182 women with PD-L1–negative endometrial carcinomas. We also studied the predictors of overall survival in the cohort. We found that increased PD-L1 expression was associated with MLH1/PMS2 loss but not MSH2/MSH6 loss. In women with endometrioid adenocarcinomas, we found that PD-L1 expression was associated with FIGO grade; poorly differentiated tumors were more likely to express PD-L1. We further examined the small subset of MLH1/PMS2–deficient grade III endometrioid adenocarcinomas and found that PD-L1 was expressed in the majority. Finally, we found a set of characteristics that independently predicted a worse overall survival—namely age older than 65 years at definitive surgery, no treatment with radiotherapy, distant metastasis, relapse, and mutant p53. Importantly, PD-L1 expression was not associated with overall survival.

In a previous study, Li et al., showed that MSH2 or MSH6 loss, MLH1 and/or PMS2 loss, or loss of any MMR protein was associated with increased PD-L1 expression [12]. In contrast, we found that increased PD-L1 expression was associated with MLH1/PMS2 loss but not MSH2/MSH6 loss. MSH2/MSH6 loss is usually caused by germline mutations; however, other causes are possible, including germline mutations in EPCAM, an inversion of MSH2 (exons 1–7), and somatic mutations of MSH2/MSH6 [13]. If these mechanisms differentially affect PD-L1 expression, we speculate that the mechanisms underlying MSH2/MSH6 loss in our sample may be different from the mechanisms underlying MSH2/MSH6 loss in the sample described by Li et al. [12]. However, only a minority of cases of MSH2/MSH6 loss are related to mechanisms alternative to germline mutations. More likely, the discrepancy may be related to our measurement methods; Li et al. measured PD-L1 expression in both tumor and immune cells, while we measured PD-L1 expression in tumor cells only. Interestingly, Pasanen et al. showed that MMR deficiency is related to PD-L1 expression in immune cells but not tumor cells [14].

We also found that PD-L1 expression was not related to isolated losses of PMS2 or MSH6. In support, Engerud et al. showed that PD-L1 expression was not related to PMS2 or MSH6 [15]. However, they did not distinguish between isolated and dual losses. This distinction is important because we found that PD-L1 expression was associated with dual MLH1/PMS2 loss but not solitary PMS2 loss. On another note, Sloan et al. found that PD-L1 was expressed in the tumor cells of eight out of eight tumors with solitary MSH6 loss [16]. In contrast, we found that PD-L1 was expressed by the tumor cells of only three out of 19 tumors with solitary MSH6 loss. This discrepancy may be related to different mechanisms underlying solitary MSH6 loss, the use of different antibody clones, or small sample sizes in both of our studies.

We studied the association between PD-L1 expression and the FIGO grade of endometrioid adenocarcinomas and found that PD-L1 expression was increased in poorly differentiated tumors. Some authors have shown the same results [12,15]. Other authors have shown no association [17,18,19]. In addition, Sungu et al. showed that the FIGO grade of endometrioid adenocarcinomas is associated with PD-L1 expression in immune cells but not tumor cells [20]. To explain the heterogeneity of results, we hypothesized that the relationship between the FIGO grade of endometrioid adenocarcinomas and PD-L1 expression may be mediated by MLH1/PMS2 loss because MMR deficiency is common in high-grade endometrioid adenocarcinomas [21]. However, we examined the relationship between FIGO grade of endometrial carcinomas and MLH1/PMS2 loss and found no significant association. Further, we measured the frequency of PD-L1 expression in six MLH1/PMS2–deficient grade III endometrioid adenocarcinomas and found that PD-L1 was expressed in four (66.7%). These findings suggest that MLH1/PMS2 loss and poor differentiation are independently associated with PD-L1 expression.

We identified several well-studied factors that portend a poorer prognosis in endometrial carcinomas, but PD-L1 expression was not a predictor of overall survival in our cohort. Previous reports on the prognostic significance of PD-L1 are at odds. On the one hand, Zhang et al. found that PD-L1 expression in tumor cells was associated with better OS in 221 women with endometrial carcinomas [22]. On the other, Gulec et al. found that PD-L1 independently predicted a worse overall survival in 53 women with type II endometrial carcinomas [23]. However, in support of our results, Lu et al. performed a recent meta-analysis of nine studies and concluded that PD-L1 expression is not associated with overall survival or progression-free survival in women with endometrial carcinomas. Nevertheless, they acknowledge that further studies are needed to validate their findings because the data available to date are limited [24].

The main strength of our study is that we performed an analysis of data from a cohort of Middle Eastern women with PD-L1–positive endometrial carcinoma—an understudied population. However, our study has several limitations. First, PD-L1 expression was measured by only one pathologist. Brunnström et al. have shown that the measurement of PD-L1 expression is subject to significant interrater variability that is unrelated to training or experience [25]. Second, we measured the expressions of MMR proteins but not the underlying mechanisms, which may differentially affect the expression of PD-L1. Third, some of our analyses may have been underpowered to detect associations because the frequencies of some characteristics were low. For example, we identified solitary PMS2 loss in only nine tumors. Many characteristics of interest are generally uncommon and may explain the heterogeneity of findings across studies. We recommend a meta-analysis to consolidate current evidence.

Tumors with MLH1/PMS2 loss (23.8% of all tumors in our sample) and high-grade endometrioid adenocarcinomas (13.5% of all endometrioid adenocarcinomas in our sample) were more likely to express PD-L1 in tumor cells. Given that PD-L1 expression may predict the response to anti-PD-1/PD-L1 monoclonal antibodies in endometrial cancer, further research is required to investigate whether the presence of either characteristic signals a higher likelihood of a favorable response if immunotherapy is administered.

## Figures and Tables

**Table 1:** List of immunostains used.

Antibody	Clone	Retrieval	Concentration	Company
MLH1	M1	Heat 90	Ready-to-use	Roche
PMS2	EPR3947	Heat 90	Ready-to-use	Roche
MSH2	G219-1129	Heat 60	Ready-to-use	Roche
MSH6	44	Heat 60	Ready-to-use	Roche
p53	DO7	Heat 64	Ready-to-use	Roche
PD-L1	SP263	Heat 64	Ready-to-use	Roche

**Table 2:** Clinical and tumoral characteristics of *n* = 231 women with endometrial carcinomas overall and stratified by PD-L1 expression.

Characteristic	Total, *n* = 231 (%)	PD-L1–Negative, *n* = 182 (%)	PD-L1–Positive, *n* = 49 (%)	*p*-Value
Age group at definitive surgery				0.87
≤65 years	92 (39.8)	72 (39.6)	20 (40.8)	
>65 years	139 (60.2)	110 (60.4)	29 (59.2)	
Nationality				0.84
Jordanian	200 (86.6)	158 (86.8)	42 (85.7)	
Other	31 (13.4)	24 (13.2)	7 (14.3)	
Family history of cancer				0.98
No family history	133 (57.6)	108 (59.3)	25 (51.0)	
Second-degree relative	10 (4.3)	8 (4.4)	2 (4.1)	
First-degree relative	45 (19.5)	36 (19.8)	9 (18.4)	
Missing	43 (18.6)	30 (16.5)	13 (26.5)	
Histopathology				0.32
Endometrioid adenocarcinoma	156 (68.1)	126 (69.2)	30 (61.2)	
Grade I	52 (33.3)	46 (36.5)	6 (20.0)	
Grade II	83 (53.2)	68 (54.0)	15 (50.0)	
Grade III	21 (13.5)	12 (9.5)	9 (30.0)	
Serous carcinoma	33 (14.4)	24 (13.2)	9 (18.4)	
Carcinosarcoma	17 (7.4)	15 (8.2)	2 (4.1)	
Undifferentiated/dedifferentiated carcinoma	9 (3.9)	7 (3.8)	4 (8.2)	
Mixed cell adenocarcinoma	10 (4.3)	6 (3.3)	4 (8.2)	
Clear cell carcinoma	4 (1.7)	4 (2.2)	0 (0)	
Bokhman type				0.29
I	156 (67.5)	126 (69.2)	30 (61.2)	
II	75 (32.5)	56 (30.8)	19 (38.8)	
Chemotherapy				0.062
No	166 (71.9)	136 (74.7)	30 (61.2)	
Yes	65 (28.1)	46 (25.3)	19 (38.8)	
Radiotherapy				0.28
No	61 (26.4)	51 (28)	10 (20.4)	
Yes	170 (73.6)	131 (72)	39 (79.6)	
FIGO stage				0.78
I	121 (52.4)	95 (52.2)	26 (53.1)	
II	46 (19.9)	38 (20.9)	8 (16.3)	
III	42 (18.2)	33 (18.1)	9 (18.4)	
IV	21 (9.1)	15 (8.2)	6 (12.2)	
Missing	1 (0.4)	1 (0.5)	0 (0)	
Distant metastasis				0.57
No	199 (86.1)	158 (86.8)	41 (83.7)	
Yes	32 (13.9)	24 (13.2)	8 (16.3)	
Relapse				0.96
No	169 (73.2)	133 (73.1)	36 (73.5)	
Yes	62 (26.8)	49 (26.9)	13 (26.5)	
MLH1/PMS2 expression				0.044
Retained	176 (76.2)	144 (79.1)	32 (65.3)	
Lost	55 (23.8)	38 (20.9)	17 (34.7)	
Solitary PMS2 loss				0.94
No	222 (96.1)	175 (96.2)	47 (95.9)	
Yes	9 (3.9)	7 (3.8)	2 (4.1)	
MSH2/MSH6 expression				0.59
Retained	216 (93.5)	171 (94.0)	45 (91.8)	
Lost	15 (6.5)	11 (6.0)	4 (8.2)	
Solitary MSH6 loss				0.55
No	212 (91.8)	166 (91.2)	46 (93.9)	
Yes	19 (8.2)	16 (8.8)	3 (6.1)	
MMR status				0.090
Proficient	142 (61.5)	117 (64.3)	25 (51.0)	
Deficient	89 (38.5)	65 (35.7)	24 (49.0)	
p53 status				0.78
Wild-type	122 (52.8)	96 (52.7)	26 (53.1)	
Mutant	106 (45.9)	85 (46.7)	21 (42.9)	
Missing	3 (1.3)	1 (0.5)	2 (4.1)	

**Table 3:** Overall survival (OS) analysis of *n* = 231 women with endometrial carcinomas. The Cox regression model is based on 228 complete cases.

Characteristic	Kaplan–Meier Analysis			Cox Regression	
	1-Year OS Rate, % (95% CI)	5-Year OS Rate, % (95% CI)	*p*-Value	HR (95% CI)	*p*-Value
Age group at definitive surgery			<0.001		<0.001
≤65 years	93.5 (89.5–97.7)	73.3 (66.3–81.0)		Reference	
>65 years	80.4 (72.7–89.0)	50.6 (41.3–62.0)		2.44 (1.56–3.82)	
Bokhman type			<0.001		0.056
I	94.9 (91.5–98.4)	77.4 (71.1–84.3)		Reference	
II	74.7 (65.4–85.2)	36.8 (27.3–49.7)		1.77 (0.98–3.17)	
Chemotherapy			<0.001		0.49
No	90.4 (86.0–95.0)	72.1 (65.5–79.3)		Reference	
Yes	83.1 (74.4–92.7)	44.3 (33.7–58.3)		0.81 (0.45–1.46)	
Radiotherapy			0.13		0.005
No	70.5 (59.9–82.9)	55.6 (44.3–69.6)		Reference	
Yes	94.7 (91.4–98.1)	67.4 (60.7–74.9)		0.47 (0.28–0.80)	
Distant metastasis			<0.001		0.001
No	95.0 (92.0–98.1)	71.1 (65.0–77.7)		Reference	
Yes	46.9 (32.4–67.8)	21.9 (11.4–42.1)		2.48 (1.44–4.26)	
Relapse			<0.001		<0.001
No	92.9 (89.1–96.9)	82.1 (76.5–88.1)		Reference	
Yes	75.8 (65.9–87.3)	16.1 (9.1–28.5)		7.3 (4.5–11.9)	
MMR status			0.28		0.073
Proficient	87.3 (82.0–93.0)	61.6 (54.1–70.2)		Reference	
Deficient	89.9 (83.8–96.4)	68.5 (59.5–78.9)		1.70 (0.95–3.04)	
p53 status ^1^			<0.001		0.013
Wild-type	95.1 (91.3–99.0)	77.7 (70.6–85.5)		Reference	
Mutant	80.2 (72.9–88.1)	47.8 (39.1–58.4)		2.08 (1.16–3.73)	
PD-L1 expression			0.92		0.54
Negative	86.8 (82.0–91.9)	64.7 (58.1–72.0)		Reference	
Positive	93.9 (87.4–100.0)	62.7 (50.4–78.0)		0.84 (0.49–1.45)	

^1^ We excluded three cases with missing data. Abbreviations: HR, hazard ratio; CI, confidence interval.

## Data Availability

The data presented in this study are available on request from the corresponding author.
